# High heteroplasmy is associated with low mitochondrial copy number and selection against non-synonymous mutations in the snail *Cepaea nemoralis*

**DOI:** 10.1186/s12864-024-10505-w

**Published:** 2024-06-13

**Authors:** Angus Davison, Mehrab Chowdhury, Margrethe Johansen, Wellcome Sanger Institute Tree of Life programme, Marcela Uliano-Silva, Mark Blaxter

**Affiliations:** 1 School of Life Sciences, University of Nottingham, University Park, Nottingham, NG7 2RD UK; 2 https://doi.org/10.5281/zenodo.6125027; 3 Tree of Life, Wellcome Sanger Institute, Wellcome Genome Campus, Hinxton, Cambridge, Cambridgeshire, CB10 1SA UK

**Keywords:** *Cepaea*, Heteroplasmy, Mollusc, Mitochondrial DNA, Snail

## Abstract

Molluscan mitochondrial genomes are unusual because they show wide variation in size, radical genome rearrangements and frequently show high variation (> 10%) within species. As progress in understanding this variation has been limited, we used whole genome sequencing of a six-generation matriline of the terrestrial snail *Cepaea nemoralis*, as well as whole genome sequences from wild-collected *C. nemoralis*, the sister species *C. hortensis*, and multiple other snail species to explore the origins of mitochondrial DNA (mtDNA) variation. The main finding is that a high rate of SNP heteroplasmy in somatic tissue was negatively correlated with mtDNA copy number in both *Cepaea* species. In individuals with under ten mtDNA copies per nuclear genome, more than 10% of all positions were heteroplasmic, with evidence for transmission of this heteroplasmy through the germline. Further analyses showed evidence for purifying selection acting on non-synonymous mutations, even at low frequency of the rare allele, especially in cytochrome oxidase subunit 1 and cytochrome b. The mtDNA of some individuals of *Cepaea nemoralis* contained a length heteroplasmy, including up to 12 direct repeat copies of tRNA-Val, with 24 copies in another snail, *Candidula rugosiuscula*, and repeats of tRNA-Thr in *C. hortensis*. These repeats likely arise due to error prone replication but are not correlated with mitochondrial copy number in *C. nemoralis*. Overall, the findings provide key insights into mechanisms of replication, mutation and evolution in molluscan mtDNA, and so will inform wider studies on the biology and evolution of mtDNA across animal phyla.

**Supplementary Information:**

The online version contains supplementary material available at 10.1186/s12864-024-10505-w.

## Background

Textbook notions on the evolution and structure of mitochondrial DNA (mtDNA) tend not to illustrate the wide range of variation, because historical studies were limited to just a few groups, often vertebrates, which have perhaps the most stable genomic features [1]. Actually, mtDNA is more variable than frequently presumed, especially in groups such as tunicates [2, 3], and some non-bilaterian animals such as Cnidaria, Ctenophora, Placozoa, and Porifera [4], as well as plants [5]. Now, the high depth of DNA sequencing coverage that has been enabled by next-generation sequencing technologies has transformed opinion, so that even in vertebrates mitochondrial heteroplasmy is now perceived as common, including in humans [6].

Of the invertebrates, mitochondrial genomes in Mollusca are fascinating because they show wide variation in size, radical genome arrangements and are frequently hypervariable within species. They are also sometimes unusual. For example, in some bivalves doubly-uniparental inheritance means that two sex-linked mitochondrial lineages co-exist, one of which is inherited through the egg (F type) and the other via the sperm (M type). Since the two types stably segregate across generations, they accumulate a highly elevated sequence divergence [7, 8], with the M type hypothesised to be associated with sex determination [9]. Likewise, the first instance of cytoplasmic male sterility in an animal was recently discovered in the snail *Physa acuta*, mediated by a mitochondrial lineage that underwent a rapid acceleration of DNA substitution rates, affecting the entire mitochondrial genome [10, 11].

In comparison to the above examples, land snails in the order Stylommatophora are unusual because they often show very high levels of mitochondrial variation between individuals in the same species, frequently having a nucleotide diversity between individuals of 10%, and up to 30% in some instances. The assumption is that land snail mtDNA has a high rate of molecular evolution, an inference first made in the grove snail *Cepaea nemoralis* [12], and since supported by multiple studies in other snails [13–16]. Preliminary studies suggest that the rate of molecular evolution of snail mtDNA is also high relative to the rest of the genome [13, 17]. Unfortunately, these inferences are based on a relative paucity of data, with estimates of mutation rate (mtDNA *and* nuclear) lacking for many eukaryotic lineages and animal phyla [18, 19].

In recent years, significant advances have been made in understanding molluscan variation and mitochondrial evolution in general, especially since the advent of cost-effective genome sequencing methods (e.g. [9, 16, 20]). However, the mechanistic explanation for the high variation in land snail mitochondrial DNA is still not clear [21–23], whether there is a high mutation rate, low functional constraint, some combination of both, or perhaps some other explanation. There has also been a relative lack of progress in studying the mutational events that create the variation, including heteroplasmy, especially by direct observation in pedigrees and laboratory lines. Studies are scarce in comparison with the much greater progress achieved in other animal and plant groups [e.g. [5, 24], and especially in humans, for which the latter have been justified by a desire to understand disease [25].

The general lack of progress in molluscan mitochondrial biology and genomics in comparison to other phyla [with exceptions, [7, 26] is unfortunate because establishing the causative mechanism behind the high rates of variation in molluscs will enhance our knowledge of the biological history, processes and functions of animal mitochondrial genomes in general [26, 27], and specifically, mtDNA-associated disease. There is also the still important and general question of how mitochondria avoid a mutational meltdown, or at least significant declines in fitness within individuals and over generations [28]. More generally, mitochondrial genomes play a crucial role in molecular phylogenetic studies, especially in resolving relationships within Mollusca, particularly the stylommatophoran group to which land snails belong [29, 30]. Understanding the variation-producing process can lead to more precise measures of evolution rates and robust inferences of molluscan evolution.

**Fig. 1:**
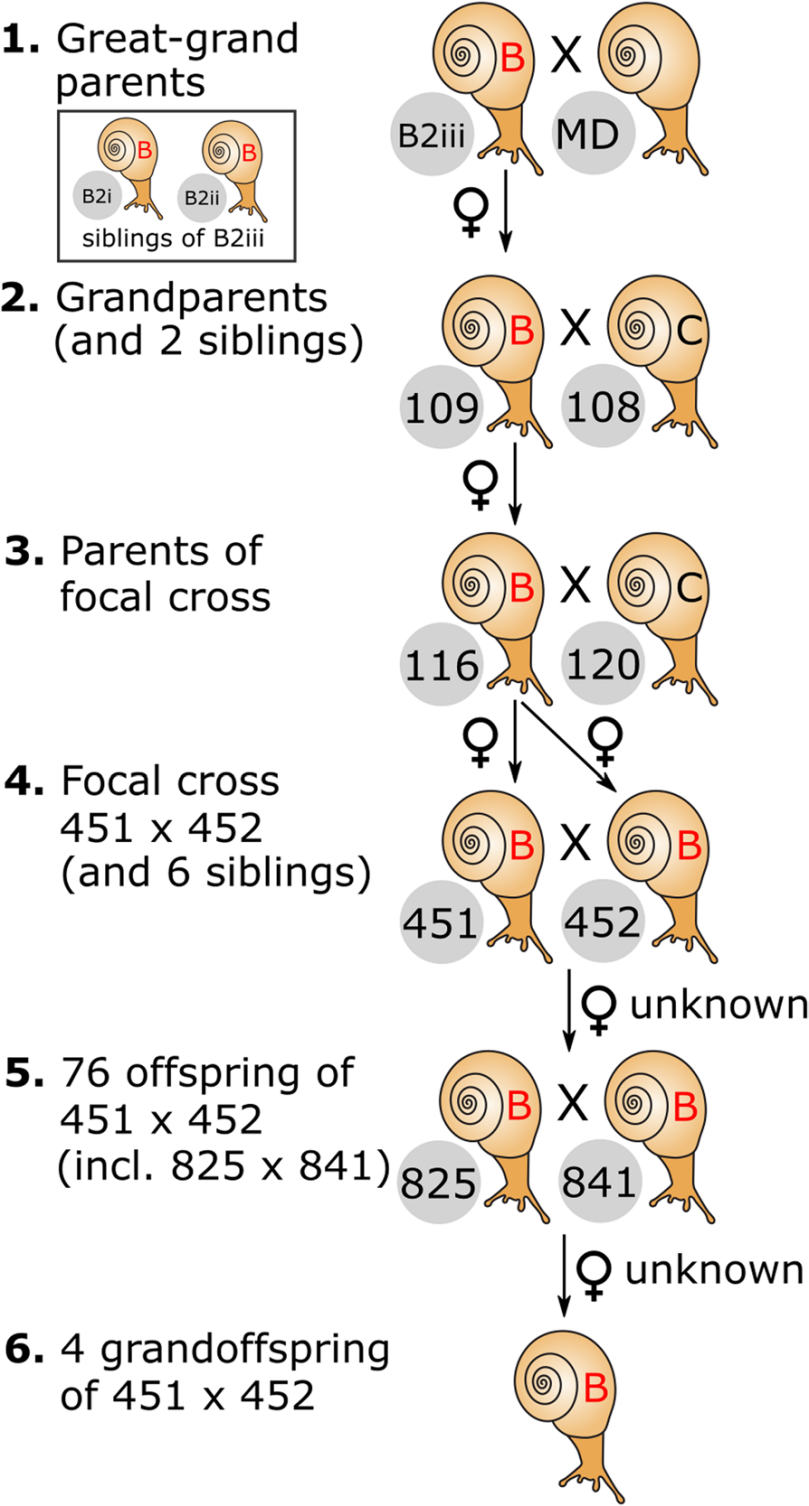
The mtDNA matriline of hermaphrodite *C. nemoralis* snails. All snails had the same mtDNA genome (lineage B, see [38]), except individuals 108 and 120 (lineage C)

*Note:* For clarity, variation in mtDNA length within an individual is referred to as “length heteroplasmy”. Variation in individual bases within the mtDNA of an individual is referred to as “SNP heteroplasmy”. Also, note that if the species is not explicitly named in the text, then the reference is to the *C. nemoralis* matriline of snails.

As part of an ongoing project to map the genes that determine colour and banding in the land snail *Cepaea nemoralis*, we generated multiple crosses that segregate for the key loci that determine variation in the patterns of the shell [31–33]. Many of these individuals underwent whole genome sequencing (WGS), using the underlying genetic variation (SNPs) and a whole genome assembly [34] to generate a chromosome-scale linkage map for the snail [35]. Here, we took the opportunity to re-use the WGS from the same crosses (Fig. 1), taking advantage of a multi-generational mtDNA matriline to understand and explore the previously unexplained length heteroplasmy in *C. nemoralis* [36, 37], as well as the origins of the extreme mtDNA divergence, including associations with mtDNA copy number, the impact of selection and evidence for germ-line transmission of SNP heteroplasmy. By also including other species, we test whether the same patterns may be widespread. The work therefore shows potential sources of variation within the mtDNA of snails, as well as informing wider studies on the biology and evolution of the mtDNA across animal phyla.

## Results

### Assembly and annotation of a reference mtDNA genome

We first assembled and annotated a reference *C. nemoralis* mitochondrial genome, using a snail from the matriline (C691, accession OP910114; Tables 1, 2; Figure S1). This genome has the expected full complement of 13 protein coding genes and 2 rRNA genes. It also has a full complement of annotated tRNA genes, unlike the original accession (U23045; [36, 37]), but with two copies of tRNA-Val (see next section). A few minor differences compared with the original submission include a longer cytochrome c oxidase subunit I (start codon 11 amino acids upstream), as well as longer versions of *ND1* (16 amino acids), *ND4L* (78 amino acids), *ATP6* (30 amino acids), and *ND4* (17 amino acids); the cytochrome b gene in the new accession is 9 amino acids shorter, with the 16S rRNA also shorter, by ~ 400 bases.

**Table 1.**
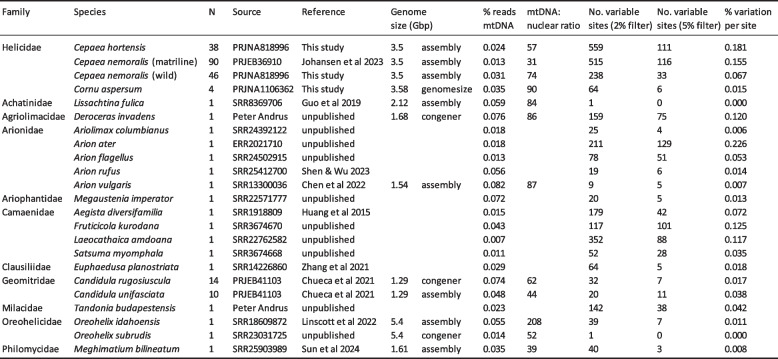
Samples used in this study. The percentage mtDNA-derived sequence reads, the inferred mtDNA to nuclear genome copy number ratio, the number of variable sites and the percent variation per site are also shown, the latter two estimated using bam-readcount [39]. Genome size estimates were not available for some species and/or we used estimates from related species, including data from genomesize.com [40]. Note that 95 snails were in the matriline but 2 were a different haplotype and 3 were sequenced separately, so were not used to estimate % reads and variation

**Table 2.**
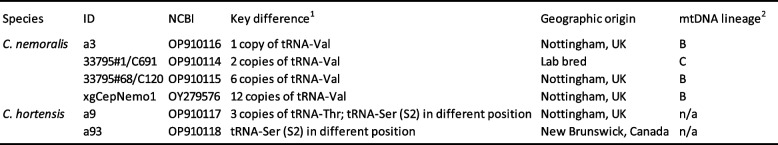
Summary of key annotation differences in reference assemblies

^1^Compared with a3

^2^See Ramos Gonzalez et al [38]

### Copy number variation of mitochondrial tRNA genes within and between individuals

The two copies of tRNA-Val in the C691 assembly are each contained within a larger 84 base pair direct repeat, separated by a spacer (with no annotated features) of approximately 160 base pairs. The reality of this repeat was corroborated by the reads mapping to form the Circos plot, which showed deeper read-depth in the tRNA-Val region (Figure S1). Blastn showed that the spacer has some imperfect hits to three contigs in the *C. nemoralis genome*, one of which has the same region repeated 11 times (JACEFZ010027795). Blastx did not reveal any similarity to any known proteins.

To investigate tRNA copy number further, we compared assemblies from 90 individuals in the same matriline, all of which should have an identical mitochondrial genome sequence, assuming an absence of de novo mutations. The 90 assemblies variously had between 2 and 6 copies of tRNA-Val, with the same spacer between direct repeats.

**Fig. 2:**
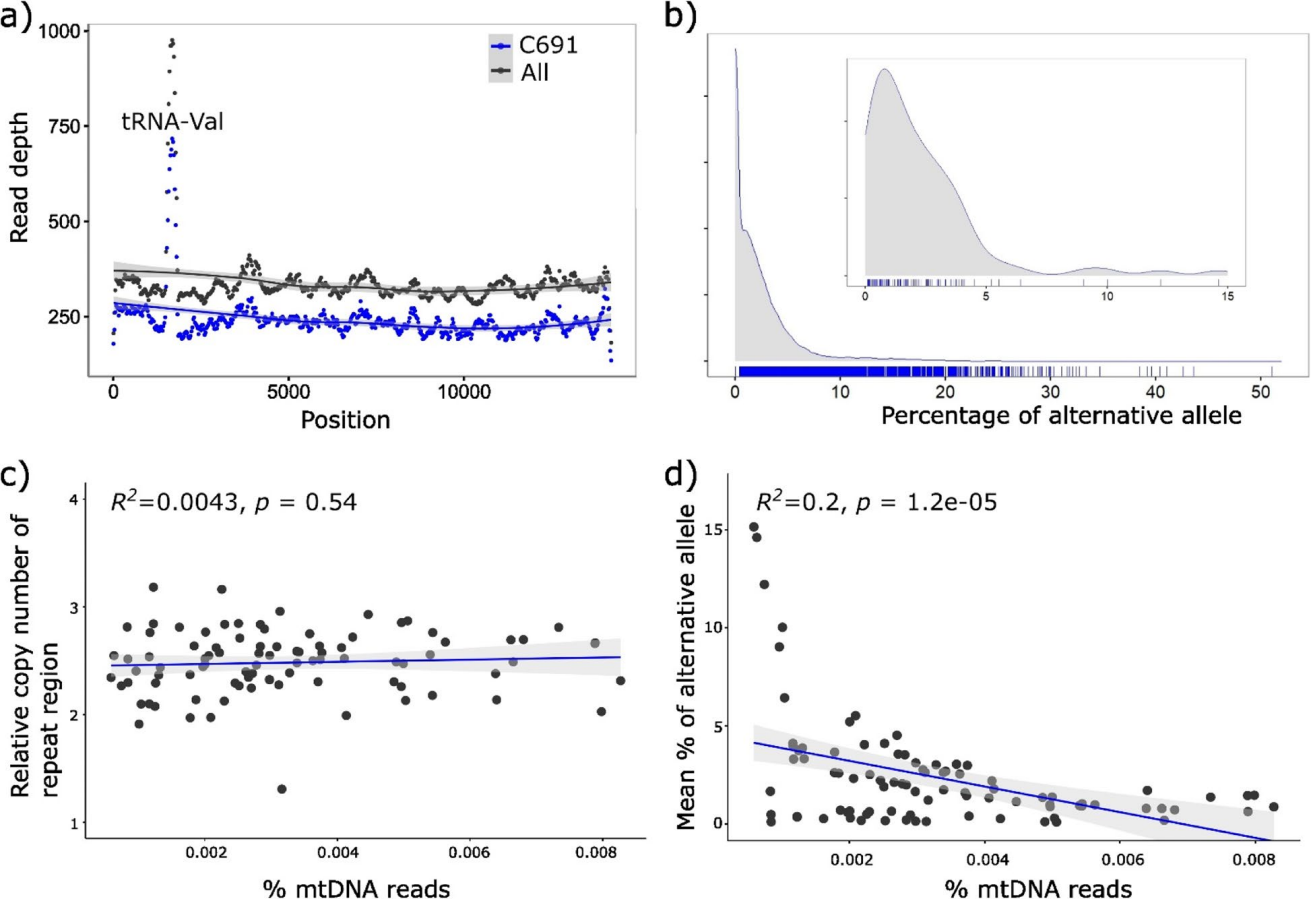
**a** Read depth across the mtDNA genome for individual C691 (blue) and the mean of all individuals (grey), using Mosdepth. Peak corresponds to tRNA-Val region. **b** Density plot showing percentage of alternative alleles in filtered VCF file. Main graph shows density plot across all sites and all individuals. Inset graph shows same plot but using the mean value for each individual. **c** and **d** mtDNA copy number (% mtDNA reads out of total) versus c) relative copy number of the repeat region and **d** mean percentage of the alternative allele for each individual

Mosdepth was used to compare the read depth of the repeated region against the read depth of 1 kb of sequence immediately upstream and downstream of the repeat, again using the C691 mtDNA genome as a reference. These analyses showed that the read depth of each of the two tRNA-Val copies (positions ~ 1510–1593, ~ 1774–1833) was one to two times greater than adjacent regions, implying around two to four copies on average per mitochondrial genome (Fig. 2a; Table S1). Consistent with this, the spacer between the tRNA-Val repeats (~ 1594–1773, one copy in the assembly) had an average depth of between two to four times that of the flanking DNA.

To understand whether length heteroplasmy is also evident in snails with other mtDNA lineages, we compared the C691 annotation against assemblies of other unrelated *Cepaea* individuals (Table 2). The mitochondrial assembly of individual *C. nemoralis* a3 (OP910116; not in the same matriline) did not have any repeated tRNA genes, but read-depth analyses showed that this is likely misleading, because there was again a pronounced increase in the read-depth in the tRNA-Val region; a repeated assembly of the same individual had five tRNA-Val copies. Another individual from the pedigree (but not in the matriline; C120) also had repeated tRNA-Val units. Finally, the mitochondrial assembly of the long HiFi reads from individual xgCepNemo1 produced an assembly with twelve copies of the tRNA-Val. Thus, we found evidence for repeated tRNA-Val units in all *C. nemoralis* individuals examined, irrespective of technology used.

In comparison, in *C. hortensis* there was no evidence of a repeated tRNA-Val in an assembly of two individuals (a9, a93, OP910117-8; Table 2). However, once again, repeated assembly using the same data sometimes produced different outcomes, including evidence that there are between 1 and 7 copies of tRNA-Thr (including a9, Table 2), with 2 copies most common (1 to 7 copies in 17, 27, 11, 9, 3, 1, 1 individuals, respectively). Inspection of read depth confirmed that there is copy number variation within individuals, as well as showing the region of the *COX3* gene may also be duplicated multiple times (Figure S1).

To investigate whether repeats might be present in other snail mitochondrial genomes, albeit not previously reported, we assembled 24 mitochondrial genomes for the snails *Candidula unifasciata* (*n* = 10) and *C. rugosiuscula* (*n* = 14), and annotated the tRNA genes. The ten *C. unifasciata* genomes all had a single tRNA-Val copy, whereas *C. rugosiuscula* had between 1 and 24 copies in the assembly (mean = 5.6, SD 5.3). In comparison, the species other than *Cepaea* and *Candidula* (Table 1) had a single copy of tRNA-Val.

### Proportion of mtDNA reads

For *C. nemoralis*, the proportion of mtDNA reads relative to the total varied about 14-fold across all matriline individuals (Table S2), from ~ 0.002% to 0.03% of reads (~ 1 in 3,000 to 1 in 45,000 reads). Similarly, in *Candidula* species, the proportion of mtDNA reads varied about eightfold, from 0.017% to 0.13%. The proportion of mtDNA reads across all species averaged around 0.036%, but ranged from 0.007% (*Laeocathaica amdoana*) to 0.08% (*Arion vulgaris*).

In subsequent analyses, we also used the raw sequencing reads to estimate relative copy number, specifically number of mtDNA copies per nuclear genome copy, because this is more intuitive in terms of biological meaning and is a better measure for cross-species comparisons. An average ratio of 31 in the *C. nemoralis* matriline was low compared to most other species (Table 1), ranging between 5 and 80 in each individual. In wild *C. nemoralis* and *C. hortensis*, the ratio ranged from 23/4 to 188/127, respectively. In other species, the highest ratio was for *Oreohelix idahoensis* (208), with most species having between ~ 30 and ~ 90 mtDNA copies per nuclear genome (Table 1).

### Elevated SNP heteroplasmy in the matriline

A high number of sites in the matriline showed evidence for SNP heteroplasmy. Specifically, 666 sites showed biallelic variation across the 90 individuals, reduced to 529 sites after Q30 filtering, and further reduced to 362 sites using a Q90 filter (batch 1). No site showed a fixed difference compared with the parental type (Table S2); instead, the majority of alternative allelic variants were at relatively low frequency (< 10%; Fig. 2b, main figure), yet also found across multiple individuals. The mean number of sites that were variable in each individual was 372 (around 2% of all sites, S.D. = 179). In two individuals the alternative allele was greater than 50% at two positions (Table S2), position 10,655 in C770 (51%, non-synonymous but conservative change I > L) and position 10,312 in C849 (52.5%, in 12S rRNA). At position 10,655, the alternative allele was absent in all other individuals except one other (C773, 0.4%); at position 10,312, the alternative allele was found in many individuals, often at high frequency.

**Fig. 3:**
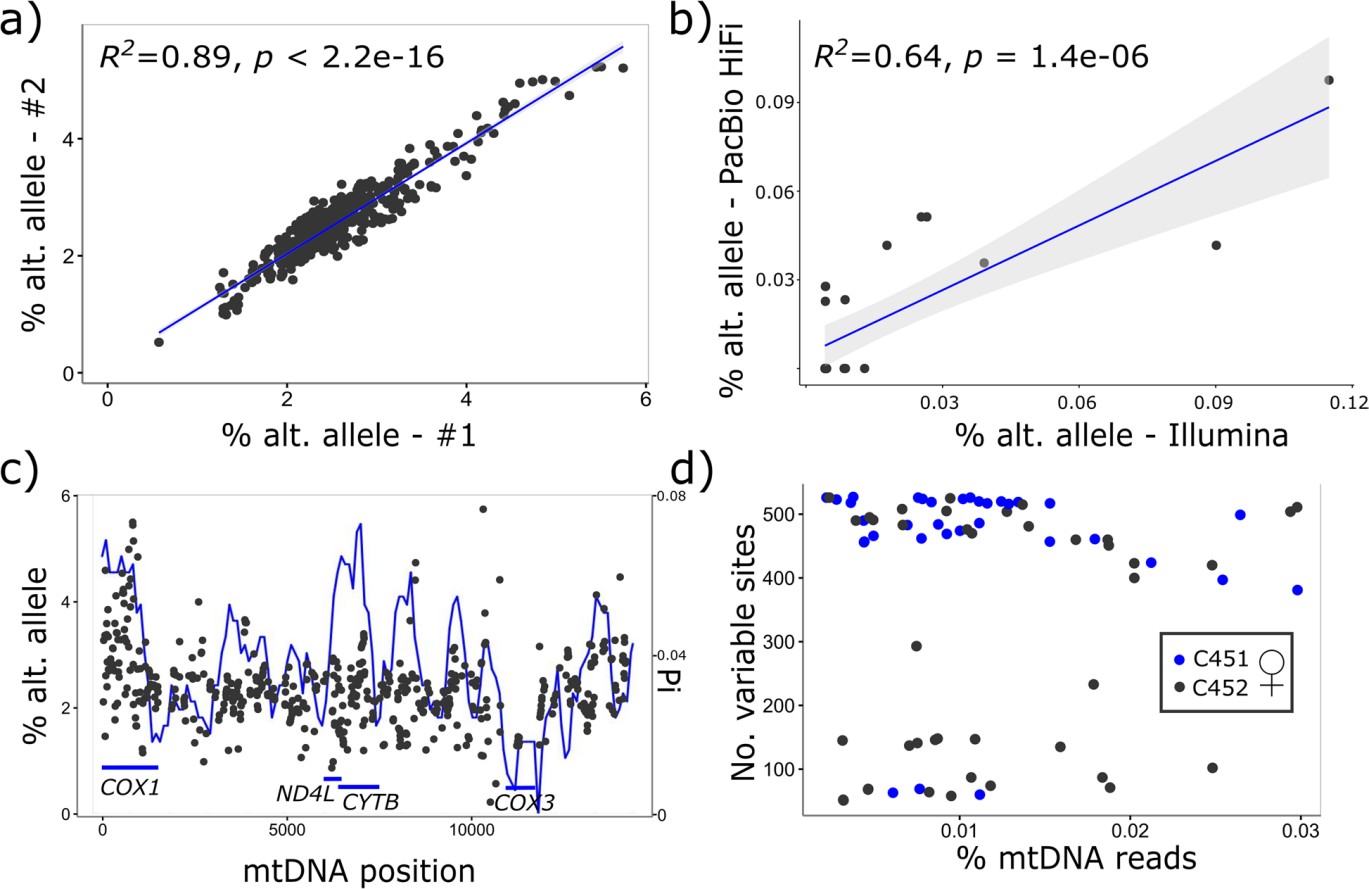
**a** Mean frequency of alternative allele in batch 1 versus batch 2 Illumina sequences **b **Frequency of alternative allele, comparing Illumina and PacBio HiFi sequencing. **c** Mean percent frequency of the alternative allele according to mtDNA position (points), and sliding window nucleotide diversity (Pi, blue line). d) Proportion of mtDNA reads plotted against the number of variable sites by inferred mother, either C451 (blue) or C452 (grey)

To check that this sequence variation was real, rather than technological artefact, the two Illumina sequencing runs were compared. No major differences were discovered. Analysis of variants in batch 2 resulted in 744 biallelic sites, reduced to 622 and 422 after Q30 and Q90 filters were applied. In total, 438 sites were in common between two batches using the Q30 filtered data. Analyses of mean sequence variation across individuals and between batch 1 and 2 show a strong correlation consistent between the two batches (Fig. 3a;* R*^2^ = 0.89, *p* < 2.2e-16).

To further validate the results, we compared the output from Illumina and PacBio technologies. Using bcftools to call variants and create a vcf file, only 25 sites were identified as variable. However, the same sites tended to be variable using either Illumina or PacBio HiFi methods (Fig. 3b;* R*^2^ = 0.64, *p* = 1.4e-6; Table S3), evidencing that the major part of the sequence variation is not due to the Illumina technology or a batch effect.

Finally, we compared variation in SNP heteroplasmy versus putative NUMT sequences. A limited number of NUMT-containing contigs were recovered from the genome assembly, but the variation between these sequences and the heteroplasmic sites of the mitochondrial assembly was not the same. Therefore, while some of the variation may be due to NUMT miscalls, it is not likely that the major signal in SNP heteroplasmy is caused by NUMTs.

### Nucleotide variation across the mtDNA is negatively correlated with mtDNA copy number within and between species

**Fig. 4:**
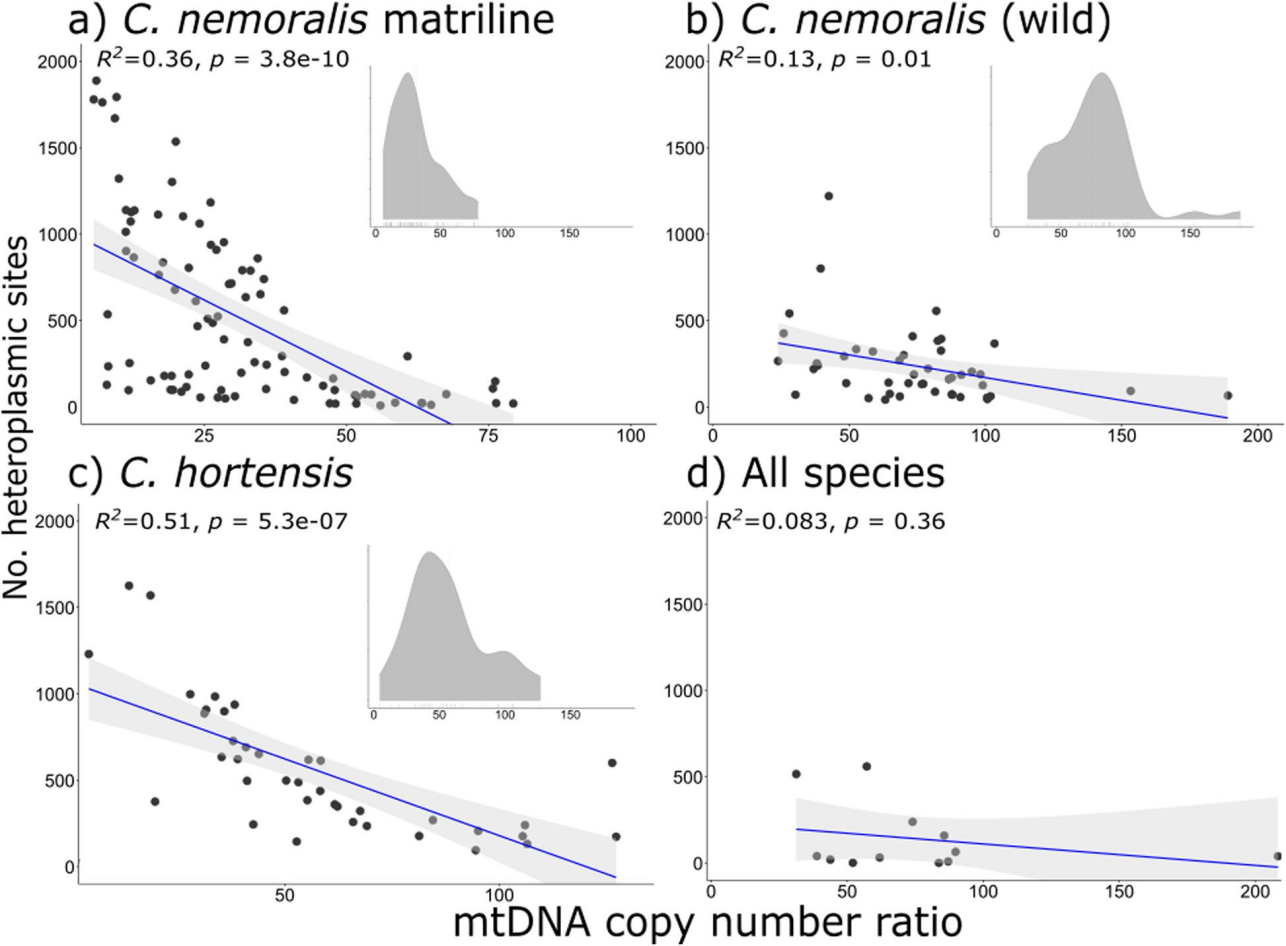
The mtDNA to nuclear genome copy number ratio versus number of heteroplasmic sites (using bam-readcount, 0.02% filter) using individuals from **a**) the *C. nemoralis* matriline **b**) wild *C. nemoralis* and **c**) wild *C. hortensis*. The inset graphs are density plots showing the mtDNA copy number ratio, lowest in the *C. nemoralis* matriline

A key finding was that percent sequence variation of each individual within the matriline of *C. nemoralis* was strongly negatively correlated with mtDNA copy number (Fig. 2d; *R*^2^ = 0.2, *p* = 1.1e-05). In comparison, repeat copy number was not correlated with mtDNA copy number (Fig. 2c). However, one issue is that variant callers do not function well when there are few individuals in a dataset, or in detecting low frequency variants. Therefore, to further test these findings, variant data were also examined by applying bam-readcount (2% filter) to the *C. nemoralis* matriline data, (Table S4), wild collected *C. nemoralis* and *C. hortensis* (Table S5), and all other species (Table S6, summary in Table 1). These analyses corroborated the initial finding. A negative and significant correlation was recovered using bam-readcount on the matriline individuals (Fig. 4a), and also using wild collected *C. nemoralis* (Fig. 4b) and *C. hortensis* (Fig. 4c). There was a negative but non-significant correlation using data from all species (Fig. 4d). Using a stricter filter with bam-readcount (5%), the associations were significant using the matriline data and the data for *C. hortensis* (Figure S2a, c).

### Distribution of sequence variation across the mtDNA genome and within genes

A few individuals showed elevated variation across multiple sites (Fig. 2b inset and Table S2). Specifically, four individuals had a mean proportion of alternative alleles (i.e. excluding zeros) in the vcf filtered dataset over 5% (including C834, C706, C857, C861), and four had means over 10% (C837, C849, C854, C855). Individuals C849 and C854 had mean alternative allele frequencies of 14–16%, including thirteen positions between them where the frequency of the alternative allele was over 30%. Using the bam-readcount output (and including all sites), the same snails showed the same pattern, with C854 having 1888/14202 (13%) sites variable, with an average frequency of the alternative allele of 1.1% (across all sites and including zeros; Table S4).

Mutations were recovered across the whole mtDNA genome, albeit with variation between regions (Fig. 3c), including elevated SNP heteroplasmy and nucleotide diversity in cytochrome oxidase subunit 1 (positions 1 to 1530), and possible reduced SNP heteroplasmy and nucleotide diversity in cytochrome oxidase subunit 3 (10,829 to 11,627). In comparison, NADH dehydrogenase 4L and/or cytochrome b showed moderate heteroplasmy but higher nucleotide diversity.

At the individual level, the majority of genes showed a strong deviation from a neutral expectation in terms of variation. For example, in comparing positions 1 and 3, cytochrome oxidase subunit 1, cytochrome b, cytochrome oxidase subunit 2, cytochrome oxidase subunit 3 and *ND4* all showed a highly significant excess of 3rd position changes, with significant changes also for *ND5* and *ND1* (Table 3). Likewise, in comparing positions 2 and 3, all of the same genes showed a highly significant excess of 3rd position changes, including also *ATP6*, ND2, *ND5* and *ND1*, with ND3 showing a significant excess. In fact, only three genes, *ND6*, *ND4L*, and *ATP8*, did not show a significant excess in comparing positions 2 and 3.

**Table 3.**
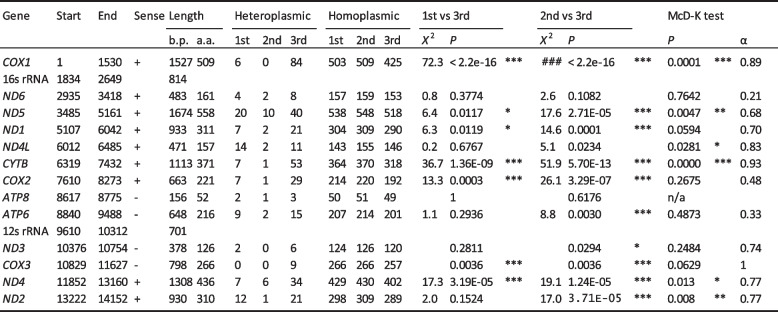
Distribution of heteroplasmic sites in C. nemoralis matriline, by codon position. The relative numbers of synonymous and non-synonymous heteroplasmic sites were also compared against an outgroup, C. hortensis, using the MacDonald-Kreitman test, also showing alpha value

Two genes showed particularly strong deviations from neutrality. For cytochrome oxidase subunit 1, there were 90 putatively variable positions, with six in codon position 1, zero in codon position 2 and 84 in codon position 3; for cytochrome b, there were 61 putatively variable positions, with seven in codon position 1, one in codon position 2 and 53 in codon position 3. Given the codon usage table, the expectation was that many position 3 changes should be synonymous, whereas position 1 mutations will tend to cause non-synonymous change, and position 2 mutations will always cause synonymous change. In keeping with this, 54/97, 28/28 and 9/334 mutations coded for non-synonymous changes in positions 1, 2, and 3, respectively. Using the McDonald-Kreitman test, six genes showed a lower ratio of nonsynonymous to synonymous variation within C. *nemoralis* compared to between species, indicating evidence for positive selection. This again included cytochrome oxidase and cytochrome b as showing the strongest deviations, as well as *ND5*, ND2, *ND4L* and *ND4* (Table 3).

### Evidence for inheritance of SNP heteroplasmy

We compared the complement of mutations shared between the sibling offspring of parents C451 x C452, with the expectation that if SNPs are shared between siblings then they are likely inherited from a common parent, except in rare cases of homoplasy. In fact, many SNPs were shared between most offspring. This is illustrated by the fact that for each variable position, an average of 53 of the 76 (70.2%, S.D. = 11.7) siblings showed variation at the same position.

We attempted to infer the putative mother of these 76 siblings, by comparing the correlation between the frequency of the variation in each parent and against each of the offspring (Table S7). Using strict criteria, we identified 27/14 snails from a C451/C452 mother, respectively. Then, using looser criteria, choosing the mother/offspring combination with the most significant association and highest *R*^*2*^ value, 50/24 snails were assigned to a putative C451/C452 mother.

**Fig. 5:**
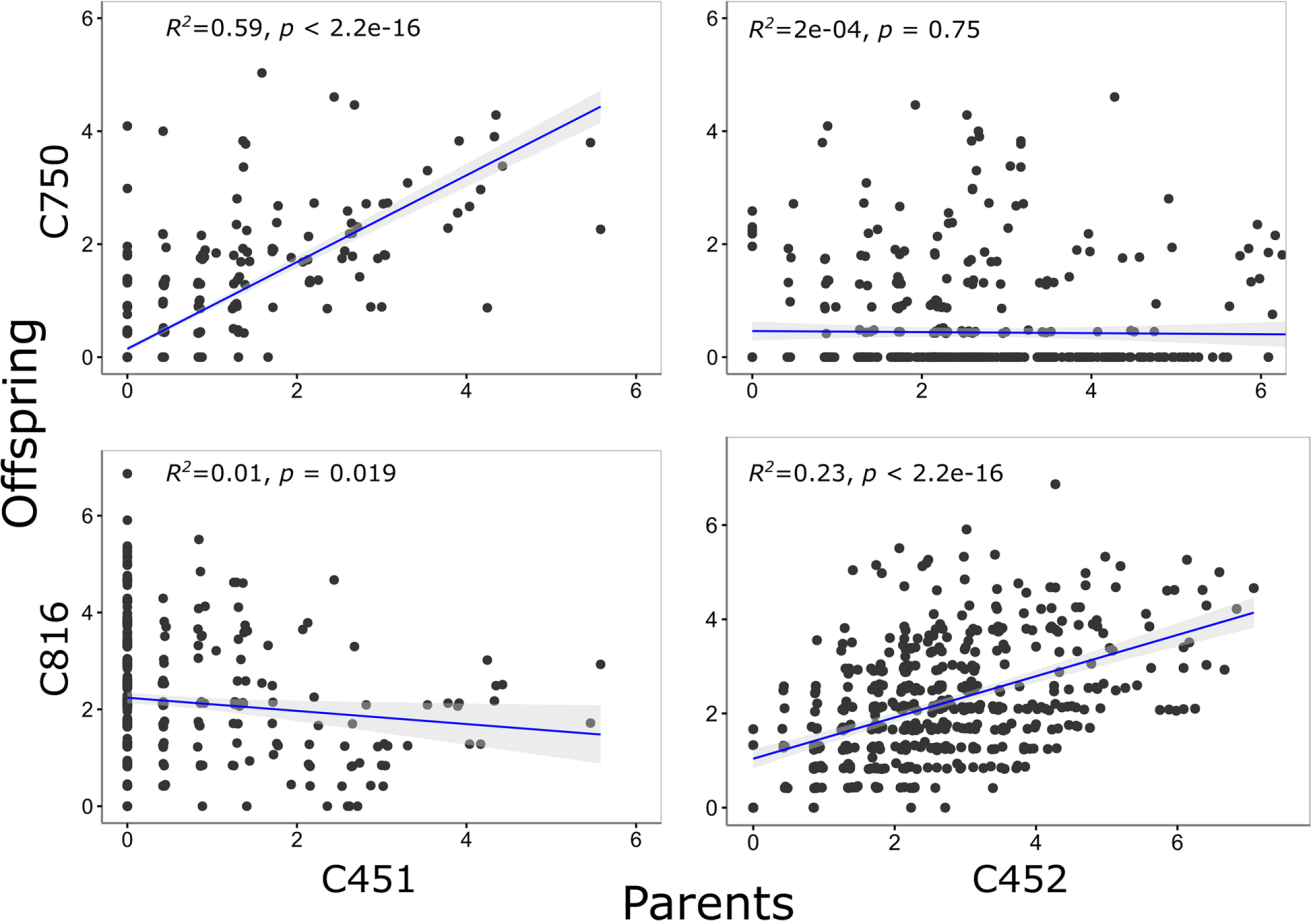
Comparisons between parent and offspring heteroplasmy plotted according to the percent frequency of the alternative allele. Putative mother, either C451 or C452, is plotted on the x axis, with offspring on the y axis

Some of these comparisons showed convincing associations, providing further evidence that the heteroplasmic mtDNA might be inherited (Fig. 5). For example, SNP heteroplasmy of C451 showed a strong positive association with SNP heteroplasmy of C750 (top left in Fig. 5a), but there was no association for C452/C750 (Fig. 5b). In comparison, SNP heteroplasmy of C452 showed a strong association with SNP heteroplasmy of C816 (Fig. 5d), but a shallow negative association between C451/C816 (Fig. 5c). These results are therefore consistent with the inference that C451 was the mother to C750 and C452 was the mother to C816.

The two parents, C451 and C452, had different levels of SNP heteroplasmy, having means of 0.4% (S.D. = 0.9) or 2.6% (S.D. = 1.3), across 124 or 520 different sites, respectively. Consistent with this, using the strict criterion to identify the mother, the maternal offspring of C452 had a higher average heterozygosity of 3.1% (S.D. = 2.1) compared to 1.3% (S.D. = 1.3) for C451 (two sample *T-*test, *P* = 0.002; Table S7). Using the more relaxed criterion there was no difference (451: 2.5% vs C452: 2.7%, S.D. 3.5, 1.9 *p* = 0.80). However, it was noted that there were two clear groups of offspring, at least in terms of numbers of SNPs observed. Putative offspring of C452 tended to fall in the higher group (Fig. 3d), again consistent with germline transmission, because C452 had higher SNP heteroplasmy.

## Discussion

We used whole genome sequencing data of the terrestrial snails *Cepaea nemoralis, C. hortensis*, and twenty other species to explore the origins of extreme mtDNA divergence, as well as previously unexplained length heteroplasmy in *C. nemoralis*. The main finding is that some individuals in the *C. nemoralis* matriline had a high proportion of mtDNA that show SNP heteroplasmy, up to 13% of all sites in one individual. Moreover, the degree of mtDNA variation in an individual snail was found to be negatively correlated with mtDNA copy number ratio, both within the matriline of snails, and also in two separate analyses using wild-collected *C. nemoralis* and *C. hortensis* (Fig. 2d; Fig. 4a-c). This was irrespective of the analysis method, whether using filtered variants or a count of the number of variants per mtDNA. It is possible that this finding may reflect a general pattern in snails but this requires further investigation, including larger samples and better knowledge of nuclear genome size (Fig. 4d). Notably, similar findings regarding mtDNA genome copy number have recently been reported in plants [5, 41].

Within individuals of *C. nemoralis* there was evidence that selection acts against deleterious mutations (Table 3), and that the variation that was measured in somatic tissue is indicative of inherited, germ-line variation (Fig. 5). In comparison, mtDNA length heteroplasmy within individuals of the *C. nemoralis* matriline, *C. hortensis* and in *Candidula rugosiuscula* was due to each mtDNA having multiple copies of tRNA genes (up to 24), likely facilitated by flanking direct repeats and error-prone replication. There was no association between the number of repeats and mtDNA copy number. The conclusion is that, while SNP heteroplasmy and length heteroplasmy may have different origins, both are indicative of a potential link between replication and mutation.

### High mtDNA SNP heteroplasmy

The *C. nemoralis* matriline individuals had a consistently high rate of SNP heteroplasmy across multiple sites, in keeping with a recent study on the New Zealand freshwater snail *Potamopyrgus antipodarum* [20], and in *Daphnia* [24]. On average, 372 or 2% of all sites showed some variation in the vcf output, with two snails having a mean alternative allele frequency of ~ 14–16% (excluding invariable sites).

Although no fixed differences or mutations were recorded between individuals within the matriline, there was otherwise a generally high rate of SNP heteroplasmy, albeit skewed so that some individuals showed an especially high rate across multiple sites (e.g. more than 10% of sites heteroplasmic). This finding was coupled with a second observation, that mtDNA genomes that are present at low copy number relative to the number of nuclear genome copies tended to have a much higher rate of reported variants.

Before accepting this finding as fact, the first issue we considered is whether the reported variants were real or else are due to sequencing error. There was no evidence that this variation is due to a technological artefact. Instead, the evidence overwhelmingly suggests that most of the SNP variants were real, perhaps discounting only those found at low frequency in a few individuals or in just one of the two batches of Illumina sequences. First, there was strong evidence for purifying selection having acted upon the variation, because the majority of putative base changes in mtDNA genes were in the 3rd codon position and/or did not cause non-synonymous changes, which can only be consistent with a biological explanation (Table 3). Second, correlations in frequency between mothers and offspring are evidence for germline transmission of the heteroplasmic sites from one generation to the next (Fig. 5), with two groups of offspring having high or low SNP heteroplasmy depending upon the putative mother (Fig. 3d); the results are consistent with the fact that somatic tissue SNP heteroplasmy reflects variation in the germline, and that this variation is sometimes transmitted to the next generation. Third, similar patterns were obtained using Illumina and long read PacBio methods (Fig. 3b), which is not always the case [20]. Finally, there is no indication that the overall findings are majorly impacted by NUMTs e.g. nuclear copies would likely not show differences in non-synonymous versus synonymous changes. Additionally, those snails with the lowest mtDNA copy number had the highest absolute number of variants, a result that can not be due to NUMTs.

In trying to make sense of these findings, caution is required because – as others have emphasised – there is difficulty in disentangling the intertwined roles of mutation, selection and drift in the mitochondrial genome [42], including bottlenecks in the germ-line [43]. Mitochondrial DNA copy number may also vary between tissues, age and is also sometimes associated with pathology [44, 45]. There is also the problem that the association between SNP heteroplasmy and copy number is, of course, just a correlation, and does not necessarily imply causation and/or directionality.

One interesting aspect is that if the findings are taken at face value then it could be deemed that they counter a conventional wisdom of biology, that mtDNA mutations are only “important” and have a phenotypic effect when they exceed a relatively high threshold level, typically cited as greater than ~ 60–80% of mutant versus wild-type [27, 46–48]. The thinking is that novel (especially mildly deleterious) alleles can rise to mid–low frequency within a cell, yet still not be visible to selection. If this theory is correct, then most SNP heteroplasmy should represent either haploinsufficient or recessive mutations, because of the high mtDNA copy number per cell/organelle. SNP heteroplasmy where the alternative allele is at high frequency tends to be associated with a significant excess of nonsynonymous mutations [49].

Yet in comparison we found that alternative alleles at highest frequency were synonymous changes, implying that selection had removed non-synonymous changes when the novel allele was still at low frequency. In support of this finding, there was no evidence that relative mtDNA copy number was associated with higher or lower levels of non-synonymous mutations (not shown). The conclusion, therefore, is that selection must have acted against mutational changes (especially non-synonymous), even when alleles were at low frequency and irrespective of mtDNA copy number. This is in keeping with findings that have compared male-transmitted and female-transmitted mtDNAs, associated with DUI in bivalves, and the impact upon selection [50].

However, there is actually no disparity between this and other work, because studies that demonstrate an impact of SNP heteroplasmy on the phenotype have been largely centred on disease-associated variation, involving mutations that have reached a high frequency, and have not been removed by natural selection. In comparison, most non-synonymous mutations are removed when they are at low frequency, except for those that have some sort of advantage and a measurable phenotype, which then become the object of study. There is an ascertainment or study bias.

These findings may have implications for understanding the generally high rates of variation that are frequently reported in land snails, for which two explanations would be that there is either a high mutation rate and/or a low functional constraint [21–23, 28]. In our study, the evidence is consistent with selection against non-synonymous mutations. Thus, while there was lots of variation in the pedigree, as judged by the degree of SNP heteroplasmy, only two of the variants broke the threshold to reach a majority. Whatever the rate of evolution is in snails, the “pedigree rate” must exceed the “phylogenetic rate” of mutation, as has been shown in multiple other studies [28]. The more open question is to try to understand whether the base rates of SNP heteroplasmy, caused by mutation, are high relative to other species. Unfortunately, there is a problem in knowing the answer because most studies tend to focus on the few SNPs that reach high frequency, ignoring potentially high levels of “background” variation. Certainly, the numbers are high compared to humans where ∼90% of individuals may carry heteroplasmy, but mostly at very few sites (~ 1 per genome) [6]. A high rate of heteroplasmy will presumably translate to a high rate of evolution, even after filtering by natural selection.

### High mtDNA SNP heteroplasmy is associated with low mtDNA copy number

Multiple studies, largely in humans, have aimed to disentangle the factors that determine and link rates of heteroplasmy and mtDNA copy number, including for example developmental stage, tissue type and age. In comparison, there have been no studies at all in snails or even molluscs.

In some studies, mtDNA copy number has been shown to decline linearly with age in humans (though not always and not in all tissues). In comparison, heteroplasmy tends to accumulate with age, especially after 70 years [44]. Heteroplasmy in key mtDNA genes has been linked to variation at other chromosomal loci [44]. Similar to this study, mtDNA copy number was negatively correlated with the total number of heteroplasmic sites in human skeletal muscle, including two sites in particular [45]. Others have found no correlation between mtDNA abundance and protein-based mitochondrial content [51, 52], which could be that mtDNA is not limiting and so perhaps not of much functional significance. Filograna et al. [53] report evidence that suggests that high absolute copy number may counteract deleterious mutations, so that the proportion is less important.

In our study, one explanation for the association between mtDNA copy number and heteroplasmy could be that a ratcheted accumulation of deleterious mutations means that the mtDNA replicates more slowly and so does not reach the same copy number; mutations “cause” the low copy number. Alternatively, it could be that slow replication is indicative of a wider malaise in the cells, which then causes replication errors and mutation. Another important consideration is that low copy number will also cause a narrower bottleneck in the germline [50, 54], thus increasing the probability of fixation. The drift-barrier hypothesis predicts a negative relationship between synonymous substitution rates and N_e_ in both nuclear and mitochondrial genomes [55]. Perhaps, this latter explanation is the most likely?

Of course, a final alternative is that the relationship is not causative, but if so then how are they associated? Aging is the most obvious explanation, either because of variation in the age of sampled snails or tissue. This explanation is consistent with evidence in other animal groups but there are no data at all in molluscs. In this study, we did not record the age of the snail but this is probably not the main factor; the parents were kept alive much longer than the offspring (to maximise the number of offspring they would produce), yet did not show elevated rates of SNP heteroplasmy.

### Rates of mutation and patterns of variation between genes

We found generally high levels of heteroplasmy in the foot tissue of the snails, alongside evidence that this somatic variation is representative of maternal germline variation, which is inherited across generations (Fig. 5). These findings suggest that the reported high between-individual variation of *C. nemoralis* [12] likely arises because of a high baseline mutation rate. In addition, since much of the variation was contained in a few individuals, could it be that much of the standing variation originates in just a few individuals? Whichever the explanation, it is perhaps notable that recent a mitochondrial phylogeny of the Stylommatophora put *C. nemoralis* on a long branch compared with all other related species [56, 57], which is consistent with our data. Perhaps *Cepaea* really is exceptional in terms of mitochondrial evolution?

It should be possible to use the data to derive a simplified estimate of the short-term mutation rate. If it is assumed that the two de novo mutations that breached 50% were to become fixed, then an estimate is 2/(90 × 14,000) = 1.5 × 10^–6^ mutations per base per generation. While this rate is more rapid than rates used in the literature, this is expected because the comparison is between the “pedigree rate” and the “phylogenetic rate”; in other species it has been observed that there is an order of magnitude disparity between mutation rates measured over a few generations in studies of pedigrees or laboratory mutation-accumulation lines, and lower substitution rates measured over longer time frames [58, 59]. This is because selection tends to remove deleterious mutations from populations over generations, resulting in lower long-term rate estimates. Thus, for studies of molecular evolution, in snails and in any other species, it remains a better strategy to use an appropriately timed age calibration point, derived from e.g. the fossil record, a geological event or perhaps more recent archaeological evidence [60–62]. In this specific case, there is also the complication that *C. nemoralis* may have a much faster rate of mtDNA evolution than even closely related species.

Finally, there is further interesting detail in the data for each gene. Some but not all of the mitochondrial genes showed a strong deviation from the neutral expectation, an observation that can only be explained by selection acting against deleterious mutations within the foot tissue of a single individual. In particular, cytochrome oxidase subunit 1 and cytochrome b had multiple mutations in the 3rd codon position and an almost complete absence in positions 1 and 2. These results are relevant because mutational saturation is a significant problem in phylogenetics [63–68], yet at the same time cytochrome oxidase subunit 1 and cytochrome b are perhaps the two mtDNA genes most commonly used for molluscan phylogenetics. In comparison, there is an apparent lack of constraint in the mutational patterns of *ND6*, *ND4L*, and *ATP8*, which are also the same genes that appear as the most rapidly evolving (in terms of amino acid sequence variability) in phylogenetic studies. The findings may have implications in understanding the origins of the high nucleotide diversity within land snail mitochondrial genomes, and also inform upon annotation issues and choice of marker in phylogenetic studies.

### Extensive copy number variation and heteroplasmy of tRNA genes in *Cepaea* and other snails

The individual assemblies and analyses of mitochondrial read depth showed that length heteroplasmy within a matriline is due to each mtDNA having multiple copies of tRNA-Val in *C. nemoralis*, a finding that explains the length heteroplasmy originally observed by Terrett et al. [37] in the very first land snail mitochondrial DNA assembly. The most common number of copies was between two and four, but one individual had up to twelve copies. In comparison, the few individuals of *C. hortensis* that were tested instead had multiple copies of tRNA-Thr. There was no variation in tRNA copy number in eight other land snail species examined, except in *Candidula rugosiuscula* which averaged > 5 copies of tRNA-Val, with one assembly having 24 copies. One issue is that long-read sequencing is better able to recover repetitive regions, so it is likely that further examples will be revealed in the future, as reported in the fresh-water snail *Potamopyrgus* [20].

One of the leading mechanisms that has been put forward to explain mitochondrial gene rearrangements is the tandem duplication-random loss model (TDRL), whereby genes are tandemly duplicated, and then redundant copies are removed over generations by the gradual accumulation of random mutations [69, 70]. This general view is supported by multiple papers that have been able to view a static part of the process, such as observing two narrowly divergent copies of one or several genes within a mitochondrial genome [71, 72].

In comparison, the results here show multimers proliferating within an individual to create length heteroplasmy. In keeping with conclusions reached by others, this likely arises from error-prone replication of the mtDNA, perhaps because direct repeats that flank the gene encourage slipped strand mispairing [73–77]. Yet, in this study, in *Cepaea nemoralis* and in *Candidula*, the repeat is confined to a single gene, with no individual having a discrete number of copies. It does not seem likely that the repeat is indicative of a replication origin, because the repeats are direct, not inverted, and so do not form a stem-loop structure. On the other hand, could it be that the tRNA loops act as origins of replication [78]? The complication is that the location, content and structure of replication or control regions are very variable across the major molluscan lineages, with high rates of evolutionary turnover, frequently involving transposition of tRNAs into the region [26]. More generally, repeated elements have been commonly reported in a wide range of animal mitochondria; there are some reports of similar tRNA expansions in other molluscs [79].

Given the almost identical gene order between *C. nemoralis* and *C. hortensis*, and a relatively high degree of conservation with other snails such as *Candidula*, the tRNA repeats may not have contributed to recent structural evolution in land snail mitochondrial DNA. There was also no association between repeat length and heteroplasmy or mtDNA read depth, so the repeats are likely not involved in promoting the observed high rates of nucleotide diversity across the whole mtDNA genome of snails. Nonetheless, it is important to study repeated elements, because they are generally a potential source of mitochondrial re-arrangements, and also deletions, with the latter contributing to aging [80, 81]. The mutagenic potential of direct and inverted repeats is also negatively correlated with mammalian lifespan [82, 83]. Of course, these issues and are very poorly studied in animals outside a few vertebrates and other models.

## Conclusions

By providing new reference mitochondrial genome assemblies for the species *C. nemoralis*, this work resolves existing annotation issues, demonstrates potential sources of variation within the mtDNA of *Cepaea*, and provides resources for phylogenetic studies within the Mollusca, and the Stylommatophora. We therefore conclude that the error prone replication of mtDNA is likely causative of structural or copy number variation in the tRNA. The other form of variation, sometimes very high levels of SNP-based heteroplasmy, are associated with reduced copy number of the mtDNA in the cell. Although the direct cause is not clear, the analyses show that selection has removed much of the non-synonymous variation.

## Methods

### Prior work

We work on the land snail species *C. nemoralis* because it has long had a high profile in understanding ecological genetics, and in studies on the evolution and maintenance of the shell colour polymorphism [84]. As a result, *C. nemoralis* was one of the first molluscs for which a whole mitochondrial DNA (mtDNA) genome was assembled [36, 37]. More recently, a ~ 3.5 Gb draft whole genome of *C. nemoralis* was made available [34], which is being used to further understand the colour polymorphism in this species [e.g. 35, 82]. One outstanding issue is that the *C. nemoralis* mtDNA was not annotated in producing the new whole genome assembly [34]; the only mitogenome available is the original, with an unexplained length heteroplasmy, and dating from when assembly and annotation methods were considerably more error prone [36, 37]. Assembly of mitochondrial genomes from WGS studies of molluscs is now routine, with new studies continuing to provide revelations about the evolution of this unusual organelle [11, 26]. It is thus generally important that mtDNA accessions are revised and updated as necessary, which should include *C. nemoralis*.

### Snails

In previous work, we generated multiple crosses of *Cepaea nemoralis* that segregate for shell colour and banding loci [31], one of which was subsequently used to generate a whole genome linkage map [35]. In this study, we used the same snails used for the linkage map, including the same “focal cross” parents (C451, C452), but also including individuals from several earlier generations. Thus, the main dataset was a five generation mtDNA pedigree of 92 snails (Fig. 1), made up of two grandparents (wild-collected C108 father from Spain, C109 mother, lab-bred), one of their offspring (C116 mother) mated to a wild collected snail (C120 father, from UK), two of their offspring (focal cross parents C451, C452), and their siblings (C382, C383, C447, C662, C663, C665), plus 76 offspring of C451 x C452, and four grandoffspring. DNA from the foot tissue of these snails was all extracted using the same CTAB-based method [31], and then genome sequencing data were generated for the 92 individuals in the five generation pedigree, at the same time and using the same technology (see below).

Subsequently, several other snails were extracted using the method, and further genome sequences generated, using a different facility, in part as a check against batch effects. The snail pair that founded the pedigree were collected from Slieve Carron (Ireland) and Marlborough Downs (UK), but unfortunately DNA was not available. Instead, two offspring were used (C52, C53) of the founding Slieve Carron mother snail (Fig. 1), and also adding C50, a sibling of C109.

As mated snails were kept in pairs, and because *C. nemoralis* is a simultaneous hermaphrodite, unless egg laying is observed it is not usually possible to know which was the biological mother or father in batches of offspring, except by retrospectively examining the mtDNA sequence. Of key importance to this study, it turned out that the mtDNA of the founding individual from Slieve Carron [a lineage B mtDNA, see ref. 82] was carried through in a direct line of descent from the wild-collected founding snail over seven generations, with DNA samples and whole genome sequences available for six generations. The same mtDNA genome was thus found in all of the cross-derived snails, except the wild collected snail “fathers” C108 and C120 (both having a lineage C mtDNA). These samples are here collectively referred to as the “matriline”. The combined pedigree was therefore seven generations in depth, with six generations available for study, including 95 snails of which 93 are in the same matriline. Note that sequencing data for three matriline snails C52, C53 and C50 were generated at a later date, so 90 individuals were used in most analyses (Table 1).

For comparative purposes, we also included multiple wild-collected individuals of *C. nemoralis*, and the sister species *C. hortensis*. For some analyses, existing whole genome resequencing data of other stylommatophoran snail families was also used [85–92], including representatives from Helicidae, Achatinidae, Agriolimacidae, Arionidae, Ariophantidae, Camaenidae, Clausiliidae, Geomitridae, Milacidae, Oreohelicidae, and Philomycidae. A limitation for some of the analyses is that haploid genome size was not known for all of these species; low sequencing depth meant that a K-mer based method such as Jellyfish [93] could not be used to estimate genome size. One unexpected issue was that while there are mtDNA assemblies available for many stylommatophoran species, many based on traditional Sanger methods, raw whole genome sequencing data for the others are frequently lacking on public databases, with authors either not responding to requests or not willing to provide the data [e.g. 53, 54]. In consequence, the data available for other species was considerably less than apparent on first impression (Table 1).

### Whole genome sequencing

The genome of individual snails used in the five generation pedigree cross (92 snails, parts 2–6 in Fig. 1) was sequenced at the Wellcome Sanger Institute, using Illumina paired-end methodology (NovaSeq 6000 PE150), aiming for ~ 10 × fold haploid genome coverage, and running each sample over two lanes. For this study, most analyses were run on batch 1 of the data (“33795”), because this provided more than sufficient mtDNA read depth. However, some analyses were also carried out separately on batch 2 (“33797”) to check for batch effects, and to support some other analyses.

The genome of other *Cepaea* snails, including snails in the first generation of the pedigree (part 1 in Fig. 1) and all wild-collected *Cepaea* individuals, was also sequenced using the NovaSeq 6000 PE150 technology, but using the commercial supplier Novogene.

### mtDNA assembly and annotation

The mitochondrial genome of individual snails was assembled using the Illumina reads, NOVOPlasty v4.3.1 [94], using an appropriate seed sequence to identify starting reads. The software mitoZ v2.3 [95] was then used to provide a first pass annotation of the assemblies, and then further edited by comparing against the output of separate annotations using MITOS [96] and MITOS2 [97].

SnapGene viewer (Insight Software) software was used to visualise and manually edit the annotation. As recommended [26, 98, 99], rules for checking and manual annotation are detailed here. 1) Assume that tRNA predictions are correct. 2) Protein coding genes (PCG) assumed to begin at the first eligible in-frame start codon, that nearest to the preceding gene without overlap. 3) PCGs and tRNA do not overlap, so gene lengths adjusted to account for this. 4) PCGs may overlap but only if in different reading frames. 5) Stop codons are frequently abbreviated, a terminal T plus AA polyadenylation. 6) Alternative start codons may be ATA (M), ATY (I), TTG (L), GTG (V). 7) Boundaries of rRNA genes were those predicted by MITOS, and do not extend to flanking genes. Note that strict adherence to this scheme sometimes generate a "longer" reading frame then expected, based on existing annotations.

GB2Sequin [100] was used to output the five-column, tab-delimited feature table that is required for NCBI submission. The Circos module in mitoZ [95, 101] was used to illustrate gene elements and features, such as protein coding genes, rRNA genes, tRNA genes and sequence depth.

### Analyses of copy number and sequence variation

FASTP v0.23.2 software was used in pre-processing of fastq files, specifically to check the quality, and for adapter trimming and quality filtering of the reads [102]. Then, to explore read depth and de novo mutation in the mitochondrial genomes, the reads in the fastq files were aligned to the genome of one individual designated as a reference (C691), using the Burrows-Wheeler Alignment method [103] in bwa v0.7.17-r1188, with default settings and marking low-quality alignments (-M). Samtools v1.11 was then used to sort and compress the sam files to bam files. Picard tools v2.21.6-Java-11 [104] was used to remove putative duplicate reads in each bam file, then bcftools v1.10 [105] was used to call variants and create a vcf file.

To filter vcf files, vcftools was used with a missing call allowed in less than 10% of individuals, a minimum sequencing depth of between 10 and 500 reads, and a minimum Phred-scaled probability that a REF/ALT polymorphism exists at a site of 30 (1/1000, frequently used value, conservative) or 90 (1/1000000000, very conservative). The minor allele frequency was zero, of necessity because we were interested in de novo mutations which might be present in just one individual. The mean percent sequence variation of each individual within the matriline was then estimated, only including sites that showed variation (i.e. excluding invariable sites, see alternative analysis using bam-readcount and all sites, below).

For depth analyses, the software mosdepth v0.3.3 [106] was used with the bam files to estimate the mean per-base read depth across the whole genome of each individual, using a window size of 25 bp (–by 25). The proportion of mtDNA reads relative to the total number of reads in each sample was also estimated.

For the analyses of the wild collected *Cepaea* and the other species, we were interested in the heteroplasmy within an individual mtDNA, not the pairwise variation between different individuals in the same species, which of course have different mtDNA sequences. Therefore, a reference mtDNA assembly was made for each individual, using the methods described above, and then reads for that individual aligned to the respective mtDNA genome to make a bam file.

One issue is that variant callers do not function well when there are few individuals in a dataset, or, in detecting low frequency variants, such as heteroplasmy in mtDNA. Therefore, to generate unfiltered variant data for each individual, bam-readcount [39] was applied to individual bam files, using a mapping quality filter threshold of 30. The number of variable sites was then extracted using the accompanying code parse_brc.py. The data were then filtered, only retaining those sites where the alternative allele was 2% or higher, on the assumption that low-frequency variation might be due to sequencing error. As a check, in some analyses a higher threshold of 5% was used. The number of variable sites for each mitochondrial assembly and the percent variation per site were then estimated for each individual snail.

We first assessed 1) the number of mtDNA reads relative to 2) the total number of sequences generated, estimating the first from the number of retained reads in individual bam files, and the second as the total number of reads in the fastq files. In the text, this is abbreviated to “read depth”. In subsequent analyses, we also used the raw sequencing reads to estimate relative copy number (number of mtDNA copies per nuclear genome copy), because this is more intuitive in terms of biological meaning and is a better measure for cross-species comparisons. One limitation of this method is that the genome size was not known for some individuals, or is only known in a related genus or family member; in consequence. Of course, there are a wide variety of methods available to estimate mtDNA copy number [107, 108], but there is no expectation that the methods used here have any systematic biases.

### Validating SNP heteroplasmy

To discover evidence for de novo mutations and/or SNP heteroplasmy in the mtDNA matriline, putative variable positions were inspected in the filtered vcf file. The assumption was that novel or alternative alleles at high frequency are likely due to mutation within the mtDNA, whereas some low frequency variants might be due to sequencing error.

To explore this variation, we investigated how the variation is distributed across the mtDNA genome, with a focus on coding regions. The rationale was that if sequence variation was due to technological error, then the proportion of variants in first, second and third codon positions of protein coding genes should not deviate from random. Alternatively, as mutations in first and second codon positions will most frequently change the amino acid, an under-representation of first/second codon position variants would be indicative of selection against non-synonymous mutations, which could only be the case if they represent biological variation. As a first analysis, we used Chi-squared methods to compare observed versus expected numbers of mutations at 1st versus 3rd, and 2nd versus 3rd codon positions. In addition, DnaSP v6 [109] was used to estimate nucleotide diversity (Pi; sliding window length 500 sites, step 100 sites), and to implement the McDonald–Kreitman test [110]. For the latter, the null hypothesis is that the ratio of nonsynonymous to synonymous variation within the *C. nemoralis* matriline (assuming fixed differences) is equal to the same ratio between species, using a representative *C. hortensis* (individual a9 as an outgroup).

One possibility is that some of the apparent heteroplasmy was due to nuclear copies of the mtDNA (NUMTs), creating false positive mutations [111]. To explore this issue, we identified putative NUMTs in the genome assembly by BLAST, and compared the variation in the coding regions between the nuclear copies and the heteroplasmic variation. Of course, a concern is that true low frequency heteroplasmic sites may be difficult to tell apart from NUMTs, because the copy number may be similar to that of the nuclear genome; to check for this issue, a further validation was to only retain high frequency (> 5%) SNPs in some analyses. Finally, in initial analyses, alignments were made against the mtDNA genome, which could in theory inflate the number of heteroplasmic sites, if NUMTs align to the mtDNA; to explore this potential issue, separate alignments and analyses used the combined nuclear and mtDNA genomes, from which the mtDNA alignment was then extracted. These analyses did not show any major differences compared with the main part, so are not discussed further.

A potential limitation is that tissue used for DNA extraction was foot, rather than germline. Somatic mutations will not be passed onto the next generation, and so are not significant in an evolutionary sense. To understand whether the observed SNPs were also likely present in the germline, and inherited, we compared the complement of mutations shared between siblings, the expectation being that if SNPs are shared between siblings then they are likely inherited from a common parent, except in rare cases of homoplasy.

For a more precise analysis, it would have been beneficial to know which snail was the mother in the 76 offspring of focal cross of C451 x C452, but we did not observe egg-laying and also both parents had an identical mtDNA. However, if there is SNP heteroplasmy in the germline, and it is transmitted to offspring by the mother, then there is an expectation of correlation between mother and offspring SNP heteroplasmy; a relationship between father and offspring mtDNA might also be expected, albeit weaker, because C451 and C452 shared a common mother (Fig. 1). Therefore, to attempt to infer the most likely mother, either C451 or C452, and to further explore the possible inheritance of SNP heteroplasmy, we computed the correlation coefficient between the frequency of the alternative SNPs (SNP heteroplasmy) in each parent and against each of the offspring. The putative mother was judged to be the parent/offspring combination that showed a positive and significant relationship for the degree of SNP heteroplasmy at each site. In cases where the association was positive and significant for both parents, the mother was assumed to have the strongest parent/offspring association (lowest *P*-value and highest *R*^*2*^ value).

A final check was also performed to check that most variants are not due to error associated with the Illumina sequencing technology. The issue is that while Illumina sequencing is robust and has a documented low rate of base mis-calls, with the NovaSeq 6000 having the lowest reported error rate [112], the accuracy of a sequencing experiment may be variable, even using the same technology [112, 113]. Thus, one individual (xgCepNemo1, from the Wellcome Sanger Tree of Life collection) was sequenced using both PacBio HiFi and Illumina (Chromium 10x) methodologies. A reference mitogenome was assembled using MitoHiFi v3 [114]. Variation was then called using Minimap2 [115] and the duplicates removed and a vcf file created as above. Variation by position was compared for each of the technologies.

### Supplementary Information

Supplementary Material 1.

Supplementary Material 2.

## Data Availability

The new assemblies associated with this work have NCBI accessions OP910114-8, with the raw sequence reads for C. nemoralis available as BioProject accession PRJEB36910 on NCBI. PacBio sequences for individual xGCepNemo1 are available under accession PRJEB63482. Further links to data used are shown in Table 1.

## Abbreviations

mtDNA: Mitochondrial DNA
NUMT: Nuclear mitochondrial DNA
PCG: Protein coding genes
SNP: Single nucleotide polymorphism
TDRL: Tandem duplication-random loss
WGS: Whole genome sequencing
