# Supplementary material for: High heteroplasmy is associated with low mitochondrial copy number and selection against non-synonymous mutations in the snail Cepaea nemoralis
Source: BMC Genomics. 2024 Jun 13;25:596. doi: 10.1186/s12864-024-10505-w (PMC11177401; doi:10.1186/s12864-024-10505-w)

**Figure S1.** Circos plots showing representative annotation of mtDNA for both *C. nemoralis* (top) and *C. hortensis* (bottom), also showing GC content (inner track, red line represents 50%) and read depth (middle track). The *C. nemoralis* assembly has a duplicated tRNA-Val region, though the depth of sequence reads indicates further repeats. The *C. hortensis* assembly has a single copy of the tRNA-thr region, though the depth of sequence reads indicates both tRNA-thr and *COX3* are likely repeated.


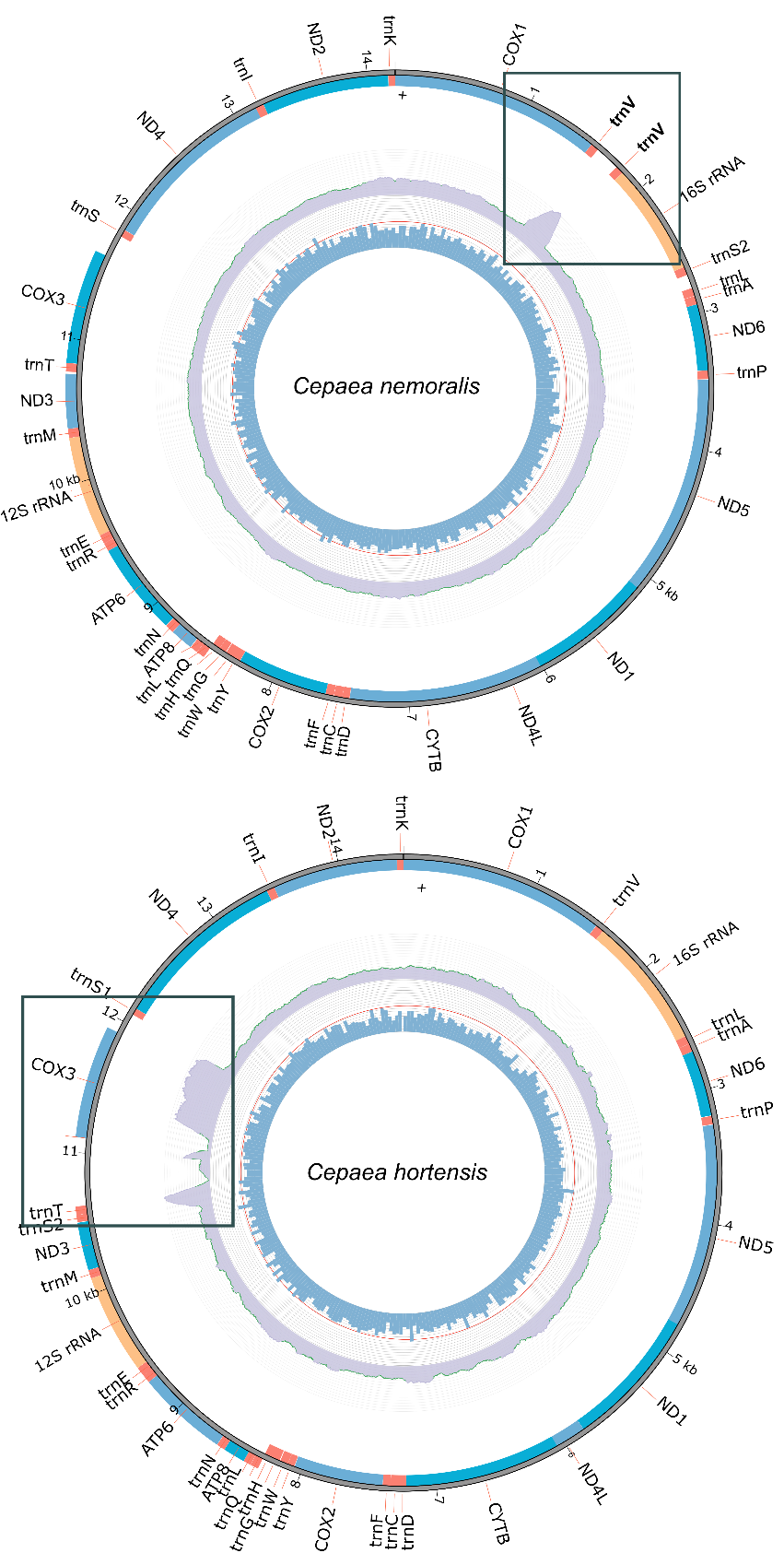


**Figure S2.** The mtDNA to nuclear genome copy number ratio versus number of heteroplasmic sites (using bam-readcount, 0.05% filter) using individuals from a) the *C. nemoralis* matriline b) wild *C. nemoralis* and c) wild *C. hortensis*.


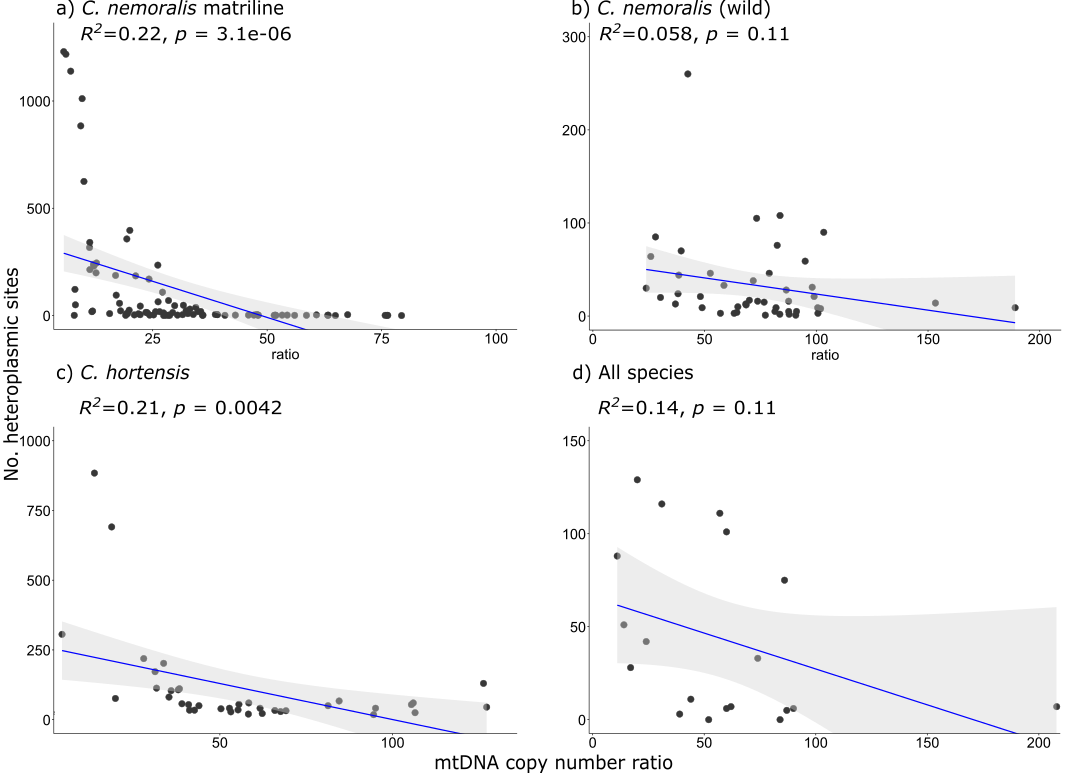

Supplement: Supplementary file 1 — Supplementary Material 1. [file 12864_2024_10505_MOESM1_ESM.docx]
